# A New Spontaneously Transformed Syngeneic Model of High-Grade Serous Ovarian Cancer with a Tumor-Initiating Cell Population

**DOI:** 10.3389/fonc.2014.00053

**Published:** 2014-03-18

**Authors:** Curtis W. McCloskey, Reuben L. Goldberg, Lauren E. Carter, Lisa F. Gamwell, Ensaf M. Al-Hujaily, Olga Collins, Elizabeth A. Macdonald, Kenneth Garson, Manijeh Daneshmand, Euridice Carmona, Barbara C. Vanderhyden

**Affiliations:** ^1^Department of Cellular and Molecular Medicine, University of Ottawa, Ottawa, ON, Canada; ^2^Centre for Cancer Therapeutics, Ottawa Hospital Research Institute, Ottawa, ON, Canada; ^3^Department of Pathology and Laboratory Medicine, University of Ottawa, Ottawa, ON, Canada; ^4^Centre de Recherche du Centre Hospitalier de l’Université de Montréal, Institut du Cancer de Montréal, Montreal, QC, Canada; ^5^Department of Obstetrics and Gynecology, University of Ottawa, Ottawa, ON, Canada

**Keywords:** high-grade serous cancer, stem cell, tumor-initiating cell, syngeneic, ovarian cancer, ovarian surface epithelium, mouse model of ovarian cancer

## Abstract

Improving screening and treatment options for patients with epithelial ovarian cancer has been a major challenge in cancer research. Development of novel diagnostic and therapeutic approaches, particularly for the most common subtype, high-grade serous ovarian cancer (HGSC), has been hampered by controversies over the origin of the disease and a lack of spontaneous HGSC models to resolve this controversy. Over long-term culture in our laboratory, an ovarian surface epithelial (OSE) cell line spontaneously transformed OSE (STOSE). The objective of this study was to determine if the STOSE cell line is a good model of HGSC. STOSE cells grow faster than early passage parental M0505 cells with a doubling time of 13 and 48 h, respectively. STOSE cells form colonies in soft agar, an activity for which M0505 cells have negligible capacity. Microarray analysis identified 1755 down-regulated genes and 1203 up-regulated genes in STOSE compared to M0505 cells, many associated with aberrant Wnt/β-catenin and Nf-κB signaling. Upregulation of *Ccnd1* and loss of *Cdkn2a* in STOSE tumors is consistent with changes identified in human ovarian cancers by The Cancer Genome Atlas. Intraperitoneal injection of STOSE cells into severe combined immunodeficient and syngeneic FVB/N mice produced cytokeratin+, WT1+, inhibin−, and PAX8+ tumors, a histotype resembling human HGSC. Based on evidence that a SCA1+ stem cell-like population exists in M0505 cells, we examined a subpopulation of SCA1+ cells that is present in STOSE cells. Compared to SCA1− cells, SCA1+ STOSE cells have increased colony-forming capacity and form palpable tumors 8 days faster after intrabursal injection into FVB/N mice. This study has identified the STOSE cells as the first spontaneous murine model of HGSC and provides evidence for the OSE as a possible origin of HGSC. Furthermore, this model provides a novel opportunity to study how normal stem-like OSE cells may transform into tumor-initiating cells.

## Introduction

Ovarian cancer is the most lethal gynecological malignancy with an estimated incidence of 238,719 cases in 2012, making it the eighth most common cancer in women worldwide (1). Epithelial ovarian cancer (EOC) is the most common type, which is further divided into endometrioid, clear cell, mucinous, low-grade serous, and high-grade serous (HGSC). HGSC is the most common and aggressive subtype of EOC, accounting for the majority of new cases (2). With a 5-year survival rate of only 40%, a greater understanding of HGSC is essential to improve patient outcome (1). The high mortality rate is due, at least in part, to a lack of screening methods to detect the disease before it metastasizes within the peritoneal cavity (3). The main reason for this inability to detect and diagnose early stage ovarian cancer is a lack of understanding of disease initiation, made even more challenging due to the current debate over the origin of HGSC. HGSC was long thought to arise from the ovarian surface epithelium (OSE) or inclusion cysts derived from them (2, 4, 5), but recent evidence has identified the distal fimbrial epithelium of the fallopian tube as the source for at least a subset of HGSC (2, 6–8).

To establish experimental models for the study of the initiation of EOC, much effort has been dedicated to the genetic modification of cells from an OSE or fimbrial origin, either in tissue culture or *in vivo*. Attempts to model HGSC have been particularly challenging and have yielded inconsistent results (5, 9, 10). Transgenic approaches have generally involved targeting specific genes known to be associated with human HGSC. This targeted approach to tumorigenesis may not be fully reflective of human disease for a number of reasons. First, it is unclear, in human disease, whether commonly mutated genes are normally involved in disease initiation and/or progression. In addition, the expression of the designed genetic changes using developmentally regulated promoters may introduce founder effects that are not reflective of human disease (9). Furthermore, it has been shown that murine cells require fewer genetic alterations than human cells to undergo transformation, again making it difficult to draw conclusions on the origin of cancer in humans from transgenic murine models (11, 12) For this reason, spontaneous models of EOC would be helpful to better understand the origins of this disease, but these models are rare and limited to the spontaneous development of ovarian cancer in hens (13, 14). New spontaneous models of HGSC are clearly needed to provide opportunities to determine the molecular basis of ovarian and fallopian tube epithelial transformation.

There is growing evidence to support the contribution of cancer stem cells (CSC) to the initiation and recurrence of cancer. The CSC theory posits that tumors arise from cells with stem-like characteristics and these cells underlie tumor heterogeneity and recurrence (15–17). Stem cells are slowly dividing cells with drug efflux mechanisms that allow them to escape the effects of chemotherapeutics that commonly target rapidly dividing cells. Another characteristic of a stem cell is the ability to generate multi-lineage progeny. Recurrent cases of HGSC maintain the heterogeneity of the original tumor suggesting that a cell with multi-lineage potential underlies tumorigenesis, instead of a single clone with a survival advantage (15). A cell with all the characteristics of CSCs is still elusive in ovarian cancer but cells with some of these CSC characteristics, identified by their expression of CD44, CD133, CD117, CD24, and ALDH1 (3), have been reported. These CSC-like cells are referred to as tumor-initiating cells (TICs) due to their increased tumorigenic capacity. The role and identification of TICs in ovarian cancer is a rapidly growing area of study.

We recently reported the first stem cell marker that identifies a subpopulation of mouse OSE cells with progenitor cell characteristics. A population of cells expressing stem cell antigen 1 [SCA1; aka lymphocyte antigen 6 complex, locus A (LY6A)] is regulated by ovulation-associated factors present in the follicular fluid and possesses a number of features of stem cells, including slow growth and capacity for self-renewal (18). After several years of establishing and growing cultures of mouse OSE cells, one cell line that was grown for a prolonged period appeared to spontaneously transform. The following body of work describes the characterization of this spontaneously transformed OSE (STOSE) cell line, demonstrating that it reliably forms syngeneic HGSC tumors. Testing of the SCA1+ cells in the parental and transformed cell lines enabled us to compare the characteristics of these stem cell-like populations, as well as determine the relative malignant potential of SCA1+ vs. SCA1− STOSE cells.

## Materials and Methods

### Experimental animals

Severe combined immunodeficient (SCID) and FVB/N mice were obtained from The Jackson Laboratory and housed with a 12 h light:12 h dark photoperiod. The animals had free access to food and water and experiments were done in accordance with the Canadian Council on Animal Care’s *Guidelines for the Care and Use of Animals*. Protocols were approved by the University of Ottawa Animal Care Committee.

### Mouse OSE cell isolation and culture

The M0505 OSE cell line was isolated and established in 2005 according to the protocol described in Gamwell et al. (18). Upon long-term passage of the cells in adherent cultures on tissue culture plates (Becton Dickinson) using MOSE media (18), the M0505 cell line spontaneously transformed and were from that point on labeled STOSE cells, which were also maintained in MOSE medium. The M1107 OSE cell line was established and maintained using the same methods as the M0505 cell line and is used as an independent control for mouse OSE cells.

### Proliferation assay

M0505 and STOSE cell proliferation was assessed from 1 to 3 days after seeding 2 × 10^4^ cells in 24-well tissue culture dishes (Becton Dickinson) in MOSE medium. The number of viable cells was determined using the Vi-CELL XR cell viability analyzer (Beckman Coulter).

### Chromosomal analysis

G-band karyotyping of 5-metaphase spreads each of M0505 and STOSE cells was carried out by the Cytogenomics and Genome Resource Facility at SickKids Hospital, Toronto, ON, Canada. Briefly, cells were harvested and colcemid (10 μg/mL) was added for 30 min and incubated at 37°C. Cells were washed, trypsinized, and a single-cell suspension was made. Following washing, a 0.075 M KCl hypotonic solution was added for 15 min and incubated at 37^o^C, and banding patterns were visualized.

### Cell cycle analysis

The percentages of cells in G1/G0, S-phase, and G2/M phases as well as the percentage of apoptotic cells were determined for M0505 and STOSE cell lines using flow cytometry. Cells were trypsinized (0.05% trypsin/0.53 mM EDTA, Corning Cellgro), washed in phosphate-buffered saline (PBS), and 1 × 10^6^ cells from three independent isolations of each cell line were resuspended in 300 μL of cold PBS. Cells were fixed in 70% ethanol for 2 h, washed, and resuspended in 250 μL of PBS and 5 μL of RNAse A (Sigma Aldrich) for 1 h. The cell suspension was then incubated for 30 min with 10 μL of propidium iodide (Sigma Aldrich) and the cell cycle was assessed by flow cytometry using a Beckman Coulter Epics XL and analyzed by ModFit LT software (Verity Software Inc.). Cell doublets were identified using fluorescence pulse height vs. area measurements and excluded from cell cycle analysis.

### Microarray analysis

RNA was extracted from M0505 and STOSE cells (*n* = 3) using RNeasy Mini Kit (Qiagen) and cDNA was made using the OneStep RT-PCR kit (Qiagen). Whole genome expression was determined using Affymetrix GeneChip Mouse Gene 1.0 ST arrays. Genes were annotated using T4-MEV software (Dana Farber Cancer Institute, Boston) and linear fold change was determined from robust multi-array average (RMA) normalized expression values. Ingenuity pathway analysis software (Ingenuity Systems, Qiagen) was used to determine functionally relevant clusters of differential gene expression. Microarray data are publicly accessible from the GEO database at record GSE54633.

### Quantitative RT-PCR

RNA was extracted using the RNeasy Mini Kit (Qiagen) and cDNA was made using the OneStep RT-PCR Kit (Qiagen). Quantitative-PCR was then performed on an ABI 7500 FAST qRT-PCR machine (Applied Biosystems) using the Taqman gene expression assay (Life Technologies) and SsoFast gene expression assay (Bio-rad). Probe (2.5 nmol) and primer (5 nmol) sequences are listed in Table 1. The level of *Tbp* was used as an endogenous control in the Taqman assay and *Ppia* was used as an endogenous control in the SsoFast assay.

**Table 1:** **Quantitative RT-PCR probe and primer sequences**.

Gene	Assay	Probe/primer sequence
*Cdkn2a*	Taqman	Probe: 5′-/56-FAM/AGCAGAGCT/ZEN/AAATCCGG CCTCAG/3lABkFQ/-3′
		Primers: forward, 5′-GCTTCAATCTGTTCCTGGCA-3′, reverse, 5′-CAACAACTTCCTCTCCTGCTAC-3′
*Sfrp1*	SsoFast	Primers: forward, 5′-CAGTTGTGGCTTTTGCATTG-3′, reverse, 5′-GAGGGAAGGGAGAGGGTTC-3′
*Frzb*	SsoFast	Primers: forward, 5′-GGACGGAGCGGATTTTCCTAT-3′, reverse, 5′-TGACAGGCTTACATTTGCAACG-3′
*Sfrp4*	SsoFast	Primers: forward, 5′-TGGAGAGATCAACTCAGTAGA AGG-3′, reverse, 5′-GGCTGGCTATCTGCTTCTTG-3′
*Ccnd1*	Taqman	Probe: 5′-/56-FAM/ATCAAGTGT/ZEN/GACCCGGA CTGCC/3lABkFQ/-3′
		Primers: forward, 5′-CGCTAGAAGTGAAGCTAAG AAGA-3′, reverse, 5′-CTTTGTGTACCGCTGGGAA-3′
*Ikbk*ε	SsoFast	Primers: forward, 5′-GGGAGAGTCTTTGCCTGATTC-3′, reverse, 5′-ATCTCCTGGGCTTGGCTATC-3′
*S100a4*	SsoFast	Primers: forward, 5′-GGAGCTGCCTAGCTTCCTG-3′, reverse, 5′-TCCTGGAAGTCAACTTCATTGTC-3′
*Spp1*	SsoFast	Primers: forward, 5′-GGAGGAAACCAGCCAAGG-3′, reverse, 5′-TGCCAGAATCAGTCACTTTCAC-3′
*Ppia*	SsoFast	Primers: forward, 5′-AGGGTGGTGACTTTACACGC-3′, reverse, 5′-GATGCCAGGACCTGTATGCT-3′
*Tbp*	Taqman	Probe: 5′-/56-FAM/ACTTGACCT/ZEN/AAAGACCATTGC ACTTCGT/3lABkFQ/-3′
		Primers: forward, 5′-CCAGAACTGAAAATCAACG CAG-3′, reverse, 5′-TGTATCTACCGTGAATCTTGGC-3′

### Intraperitoneal (IP) and intrabursal (IB) injections of STOSE cells

M0505 and STOSE cells were released from adherent cultures using trypsin (0.05% trypsin/0.53 mM EDTA), washed with PBS, and resuspended in PBS. 1 × 10^7^ M0505 cells in 500 μL of PBS were injected into the peritoneal cavity of FVB/N mice. 1 × 10^7^ STOSE cells in 500 μL of PBS were injected into the peritoneal cavity of both SCID and FVB/N mice using a 25-gauge needle (Becton Dickinson). Disease progression was monitored until humane endpoint was reached, which included 15% weight gain and/or abdominal distension. Necropsies were performed at endpoint and tumors were fixed in 10% buffered formalin for 24 h and then paraffin embedded and sectioned at 5 μm for immunohistochemical analysis.

To perform intrabursal injections of STOSE cells, FVB/N mice were anesthetized using 3% isoflurane gas and 1% oxygen. A dorsal incision was made and ovaries were externalized. STOSE cells (4 × 10^4^) were resuspended in 2 μL of PBS and injected under the bursal membrane using a 33-gauge needle and dispensing repeater (Hamilton). Tumor initiation was monitored every 2 days by palpation of the ovaries by someone blinded to the experimental groups. Disease progression was monitored until humane endpoint was reached, at which point tumors were fixed, embedded in paraffin blocks, and 5 μm sections were made for immunohistochemistry.

### Immunohistochemistry

Assessment of the histopathology of IP and IB STOSE tumors was performed by staining sections with hematoxylin and eosin (H and E) and by immunohistochemical analysis. Following deparaffinization in xylenes and rehydration in an ethanol gradient, antigen unmasking (antigen unmasking solution, Dako) was performed, followed by blocking endogenous peroxidase activity using 3% hydrogen peroxide in dH_2_O. Sections were then rinsed in PBS. Immunostaining for mouse cytokeratin (pan-CK; pre-diluted, Abcam), mouse WT1 (1:100, Dako), and mouse inhibin (1:100, Dako) was performed according to the mouse-on-mouse kit (Vector). Immunostaining for rabbit PAX8 (1:400, Santa Cruz Biotechnology) was done by incubating sections with the PAX8 antibody overnight at 4^o^C, followed by anti-rabbit horseradish peroxidase-labeled polymer (Dako) for 30 min at room temperature. All sections were counterstained using hematoxylin and developed using diaminobenzidine. Following dehydration in an ethanol gradient, sections were mounted using Permount (Fisher Scientific). Images were acquired using the ScanScope CS2 (Aperio).

### DNA sequencing

Genomic DNA was extracted from STOSE cells using QIAamp DNA Blood Mini Kit (Qiagen) and PCR amplified using custom primers designed to cover each of the 11 exons in the mouse p53 gene. Following electrophoresis on a 1% agarose gel, bands pertaining to each exon were individually excised under UV light. DNA was extracted from the agarose gel pieces using the QIAquick Gel Extraction Kit (Qiagen). Extracted DNA was then diluted to a concentration of 1 ng/μL and mixed with the appropriate custom primer (2 μM) mapping to each exon. Individual exons were sequenced using the 3730 DNA analyzer (Applied Biosystems). Sequences were aligned using the DNA Dynamo program (BlueTractorSoftware).

### Flow cytometry for SCA1 expression

M0505 and STOSE cells were trypsinized and a single-cell suspension was made using a 40 μm cell strainer. Cells were resuspended in a flow buffer (4% fetal calf serum in 1× PBS) and incubated with anti-SCA1 allophycocyanin fluorophore-conjugated antibody (Miltenyi Biotec) for 15 min at 4^o^C. Following washing and resuspension in flow buffer, cells were sorted for SCA1 expression using the MoFlo cell sorter (Beckman Coulter).

### Colony formation in soft agar

Cells were released from adherent cultures using trypsin, washed with PBS, and a single-cell suspension was achieved by passing cells through a 40-μm cell strainer. A base layer 1:1 mix of 2× Ham’s F-12:MOSE medium (Sigma Aldrich) and ultrapure LMP agarose (Life Technologies) was solidified at 4°C for 30 min and then warmed to 37°C prior to the addition of the top layer. The top layer consisting of a 1:1:1 mix of 2.5 × 10^4^ cells from single-cell suspension, 2× Ham’s F-12:MOSE medium, and ultrapure LMP agarose was added. The top layer was solidified at 4°C for 30 min and then incubated at 37°C for 7 days. Colonies were visualized using the EVOS XL imaging system (Life Technologies) and counted using ImageJ software.

### Western blot analysis

Protein was extracted from M0505 and STOSE cells using M-PER mammalian protein extraction reagent (GE Healthcare). Tumor tissue from SCA1+ and SCA1− tumors was homogenized and protein was extracted using M-PER mammalian protein extraction reagent. Protein extracts were run on a precast Nupage 4–12% bis–tris gradient gel (Life Technologies) and transferred to a nitrocellulose membrane. Following 1 h blocking in 5% non-fat milk, membranes were incubated with mouse monoclonal PAX8 (1:500, Santa Cruz Biotechnology) or mouse monoclonal P53 (1:1000, Cell Signaling) overnight at 4°C. Following washing, the membranes were incubated with rabbit anti-mouse IgG–HRP (1:5000, Abcam) for 1 h and developed using Select™ western blotting detection reagent (GE Healthcare). The same protocol was used for β-actin using mouse monoclonal anti-β-actin (1:40,000, Sigma Aldrich) and rabbit anti-mouse IgG-HRP (1:15,000, Abcam).

### Statistical analysis

All experiments were performed at least three times. A Student’s *t*-test was used to determine significant differences between two experimental conditions. Analysis of variance (ANOVA) with Tukey’s post-test was used to identify significant differences between more than two experimental groups. Statistical significance was assumed at *p* < 0.05.

## Results

### Characterization of M0505 and STOSE cell lines

**Figure 1:**
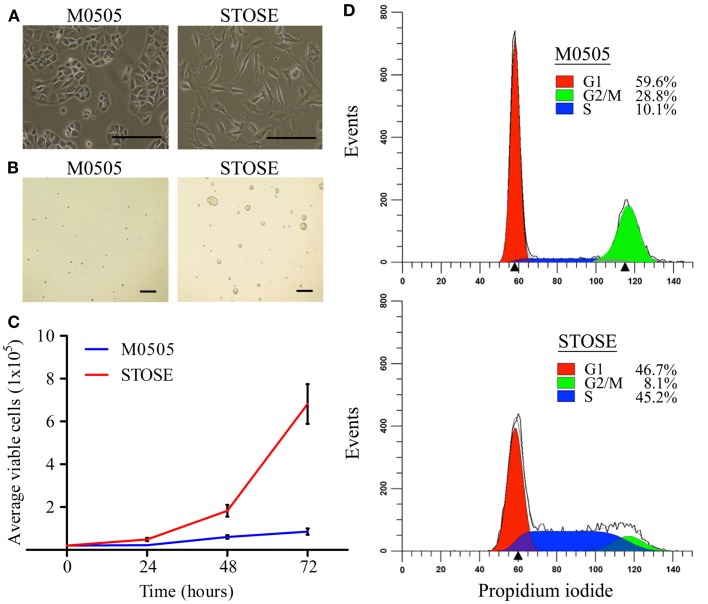
**STOSE cells exhibit classic characteristics of transformed cells**. **(A)** Bright-field microscopy of M0505 and STOSE cells. Scale bar = 200 μm. **(B)** Colony forming assay in soft agar comparing M0505 and STOSE cells. Colonies were visualized after 7 days using bright-field microscopy. Scale bar = 200 μm. **(C)** Growth curve of M0505 and STOSE cells over 3 days. Error bars represent standard error of the mean (SEM). *p* < 0.001, two-way analysis of variance. **(D)** Cell cycle analysis of M0505 and STOSE cells. Cells were incubated with the fluorescent dye propidium iodide and analyzed by flow cytometry. The average percentage of cells in G1, S, and G2/M for STOSE cells and M0505 cells is shown (*n* = 6).

**Figure 2:**
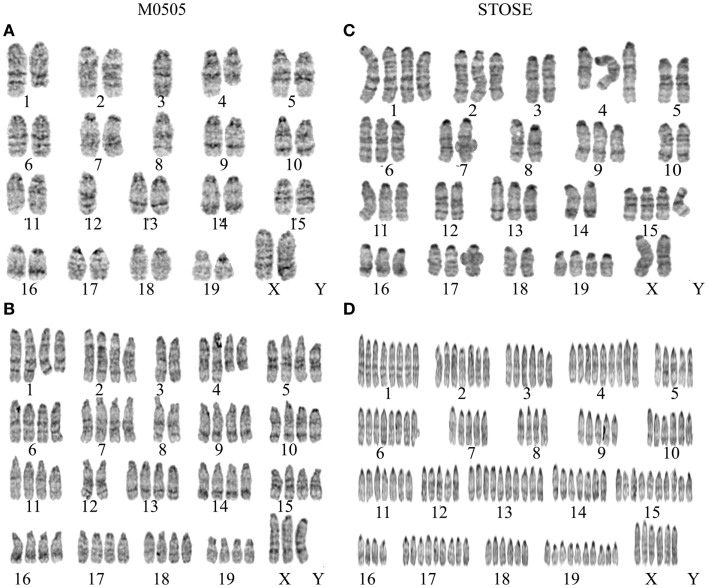
**Chromosomal analysis of M0505 and STOSE cell lines**. G-band karyotyping of five metaphase spreads was performed for both M0505 and STOSE cell lines and representative karyotypes are presented. **(A)** Near- diploid M0505 cell with 37 chromosomes. **(B)** Near-tetraploid M0505 cell with 75 chromosomes. **(A,B)** Terminal deletion of chromosomes 1 and 4 as well as loss of a chromosome 3, 8, and 12 was evident in all M0505 cells analyzed. **(C)** Near-triploid STOSE cell with 54 chromosomes. **(D)** Polyploid STOSE cell with 143 chromosomes. **(C,D)** An addition at the terminal end of chromosome 4 as well as a loss of chromosome 3, 5, and 8 were evident in all STOSE cells analyzed.

Early passage M0505 cells grow slowly, having a doubling time of 48 h. The growth rate increases as M0505 cells reach >35 passages and cells begin to lose the epithelial “cobblestone” morphology that is characteristic of early passage M0505 cells (data not shown), and has been reported by others studying spontaneous transformation of epithelial cells (19). Continual passage of late passage M0505 cells led to the establishment of the STOSE cell line. STOSE cells have lost the epithelial “cobblestone” morphology and have transitioned to a more mesenchymal morphology (Figure 1A). To determine the malignant potential of STOSE cells *in vitro*, STOSE cells were assessed for colony forming efficiency in soft agar, a measure of anchorage independent growth that is characteristic of transformed cells (20). STOSE cells formed colonies while early passage M0505 cells did not (Figure 1B). Another characteristic of transformed cells is rapid growth (20). STOSE cells have a doubling time of 13 h, almost four times faster than their untransformed M0505 counterpart. The growth rate of STOSE cells in comparison to early passage (passage 18–20) M0505 cells over 72 h is shown in Figure 1C. Since a greatly increased growth rate might be explained by aberrant cell cycle regulation, cell cycle analysis was used to determine if there were differences in the percentage of M0505 and STOSE cells in each phase of the cell cycle. Cell cycle analysis of the M0505 cells (monomers) revealed a large G1 peak (59.6 ± 1.0%), a minor S-phase population (10.1 ± 0.3%), and a surprisingly prominent, putative G2/M peak (28.8 ± 0.8%) (Figure 1D). Interestingly, the presence of a small percentage (1.5%) of hyperploid cells was detected in the analysis by the ModFit program. The presence of a small population of cells with abnormal DNA content was then confirmed by karyotype analysis that identified near-tetraploid M0505 cells (Figure 2B). In addition, the small number of diploid cells in S-phase was consistent with the observed slow proliferation of this cell line. In contrast, STOSE cells have a significantly increased proportion of cells in S-phase (45.2 ± 0.7%) and, a reduced proportion in the G1 phase (46.7 ± 0.7%). The small G2/M population and greatly increased S-phase population suggests that STOSE cell cycle checkpoints may be compromised, which could lead to the observed acceleration in the rate of proliferation.

Due to the role of aneuploidy in transformation and cancer and the abnormalities found in the cell cycle analysis, chromosomal analysis was performed on M0505 and STOSE cells to determine if aneuploidy is present. G-band karyotyping of five metaphase spreads revealed aneuploidy in both M0505 and STOSE cell lines; two representative karyotypes are shown for each cell line (Figure 2). STOSE cells have a high degree of aneuploidy with the majority of the population near-triploid (Figure 2C) and a smaller polyploid population (Figure 2D). All STOSE cells analyzed have an addition at the terminal end of chromosome 4. All near-triploid cases have a loss of chromosome 3, 5, and 8, and all polyploid cases are also hypoploid for chromosomes 3, 5, and 8 (Figures 2C,D). Surprisingly, chromosomal analysis of early passage (passage 15) M0505 cells also revealed some degree of aneuploidy with 2/5 near-tetraploid M0505 cells (Figure 2B), while 3/5 M0505 cells were near-diploid (Figure 2A). This presence of a near-tetraploid subset of M0505 cells is in agreement with the presence of M0505 cells with increased DNA content seen in the cell cycle analysis (Figure 1D). All M0505 cells analyzed have terminal deletions in chromosomes 1 and 4. All near-diploid cases have a loss of one chromosome 3, 8, and 12, and all near-tetraploid M0505 cells are hypoploid for chromosomes 3, 8, and 12 (Figures 2A,B).

### Microarray analysis of STOSE cells

To determine the molecular mechanisms by which M0505 cells transformed into the STOSE cells, whole genome microarray analysis was performed on M0505 and STOSE cells and linear fold changes were calculated for STOSE cells relative to M0505 cells. The top 10 up- and down-regulated genes in STOSE compared to M0505 cells are presented in Table 2. Interestingly, *Ddr2, Ereg, Glipr1, Calcr*, and *Ankrd1*, all up-regulated in STOSE cells, have been shown to be up-regulated in primary tumors and ovarian cancer cells (21–24). *Igfbp4* has been shown to be down-regulated in primary tumors (25, 26). The other up-regulated genes in STOSE cells: *Serpinb8, Epb41l4a, Aif1l*, and *Mgll* have no known links to ovarian cancer. Five of the 10 most down-regulated genes, *Aldh1a2, Enpp2, Lgfbp5, Thbd*, and *Uchl1*, have been previously implicated in ovarian cancer (25, 27–34). The remaining genes among these down-regulated candidates have no previous association with ovarian cancer: *Gpr64, Gpr126, Cybrd1, Star, Ncf2*. In accord with the more rapid proliferation of STOSE cells, two negative regulators of Cdk4, *Cdkn2b* and *Cdkn2a*, are down-regulated in STOSE cells 13.4- and 5.8-fold, respectively, and both *Ccna2* and *Ccnd1* are up-regulated (2.02- and 6.2-fold).

**Table 2:** **Differential gene expression in STOSE cells as compared to early passage M0505 cells**.

Gene symbol	Gene name	Linear fold change	Publications relating these genes to ovarian cancer
*Serpinb2*	Serine (or cysteine) peptidase inhibitor, clade B, member 2	90.7	Unknown
*Epb4.1l4a*	Erythrocyte protein band 4.1-like 4a	64.7	Unknown
*Ddr2*	Discoidin domain receptor family, member 2	46.4	(22)
*Aif1l*	Allograft inflammatory factor 1-like	37.8	Unknown
*Ereg*	Epiregulin	35.1	(21)
*Glipr1*	GLI pathogenesis-related 1 (glioma)	34.6	(23)
*Igfbp4*	Insulin-like growth factor binding protein 4	33.6	(25)
*Calcrl*	Calcitonin receptor-like	33.1	(26)
*Ankrd1*	Ankyrin repeat domain 1 (cardiac muscle)	30.4	(24)
*Mgll*	Monoglyceride lipase	29.8	Unknown
*Ncf2*	Neutrophil cytosolic factor 2	−61.7	Unknown
*Star*	Steroidogenic acute regulatory protein	−62.7	Unknown
*Uchl1*	Ubiquitin carboxy-terminal hydrolase L1	−70.5	(28, 32)
*Thbd*	Thrombomodulin	−76.2	(27)
*Cybrd1*	Cytochrome *b* reductase 1	−83.0	Unknown
*Igfbp5*	Insulin-like growth factor binding protein 5	−96.1	(25, 32–34)
*Gpr126*	G protein-coupled receptor 126	−96.6	Unknown
*Gpr64*	G protein-coupled receptor 64	−101.3	Unknown
*Enpp2*	Ectonucleotide pyrophosphatase/phosphodiesterase 2	−147.3	(30, 31)
*Aldh1a2*	Aldehyde dehydrogenase family 1, subfamily A2	−170.6	(29)

**Figure 3:**
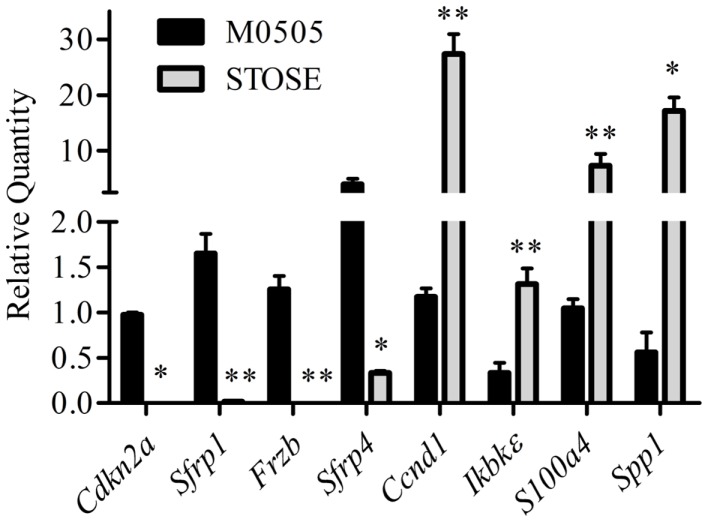
**Validation of genes differentially expressed in STOSE cells related to Wnt/β-catenin and Nf-κB signaling or in common with TCGA ovarian carcinoma arrays**. Quantitative RT-PCR analysis for *Cdkn2a, Sfrp1, Frzb, Sfrp4, Ccnd1, Ikbk*ε*, S100a4, and Spp1* expression is presented for M0505 and STOSE cells (*n* = 3). Samples used for validation are independent of those used for microarray analysis. Error bars represent SEM and *p < 0.05, **p < 0.01 by Student’s*t*-test.

The Cancer Genome Atlas (TCGA) ovarian carcinoma array is a whole genome array database with analysis of 570 human HGSC tumors. The TCGA array dataset was analyzed by the Cancer Genome Research Analysis Network and two of the top gene changes in the STOSE cell microarray were among those reported in the pathways most frequently altered in ovarian carcinomas (35): downregulation of *Cdkn2a* (−5.8) and overexpression of *Ccnd1* (+6.2). Overexpression of *Ccnd1* is strongly correlated to decreased progression free survival (36) and loss of *Cdkn2a* through mutation or hypermethylation has also been shown in human ovarian carcinomas (35, 37–39). Ingenuity pathway analysis (IPA) was used to identify functionally related clusters of gene expression differences from the microarray data. IPA analysis revealed possible aberrant Wnt/β-catenin and Nf-κB signaling in STOSE cells. The expression of multiple genes associated with Wnt signaling are significantly altered including *Cdkn2a* and downregulation of Wnt signaling inhibitors *Sfrp1* and *Frzb*. Genes differentially expressed in the Nf-κB pathway include *Spp1, S100a4, IkBk*ε, and *Ccnd1*. Interestingly, *Ccdn1* is associated with both Wnt/β-catenin and Nf-κB signaling. Validations of *Cdkn2a* and *Ccnd1*, as well as Wnt/β-catenin and Nf-κB-related genes were performed by quantitative RT-PCR on three microarray-independent samples of M0505 and STOSE cells (Figure 3).

### STOSE cells produce HGSC tumors in both SCID and syngeneic FVB/N mice

Given that STOSE cells exhibit transformed characteristics *in vitro*, their *in vivo* tumorigenicity was assessed using immunocompromised SCID mice and the syngeneic strain of mice, FBV/N. When STOSE cells (1 × 10^7^) were injected IP into four SCID mice, tumors formed in all mice (4/4) with a median endpoint of 47 days. Tumors were collected from most organs within the peritoneal cavity and the average total tumor burden was 2.22 ± 0.21 g per mouse. All SCID mice had ascites with an average volume of 5.25 ± 0.63 mL. Following IP injection of STOSE cells into immunocompetent syngeneic hosts, STOSE cells were tumorigenic in all FVB/N mice (4/4) with a median endpoint of 48 days. Necropsy revealed tumors throughout the peritoneal cavity and an average total tumor burden of 3.06 ± 0.21 g per mouse, not different from the tumors in SCID mice. All STOSE-injected FVB/N mice had ascites with an average volume of 3.08 ± 0.92 mL, also not significantly different from SCID mice (*n* = 4, *p* = 0.98). Intraperitoneal injection of 1 × 10^7^ M0505 cells into FVB/N mice did not result in tumor formation in 107 days (0/6 mice).

**Figure 4:**
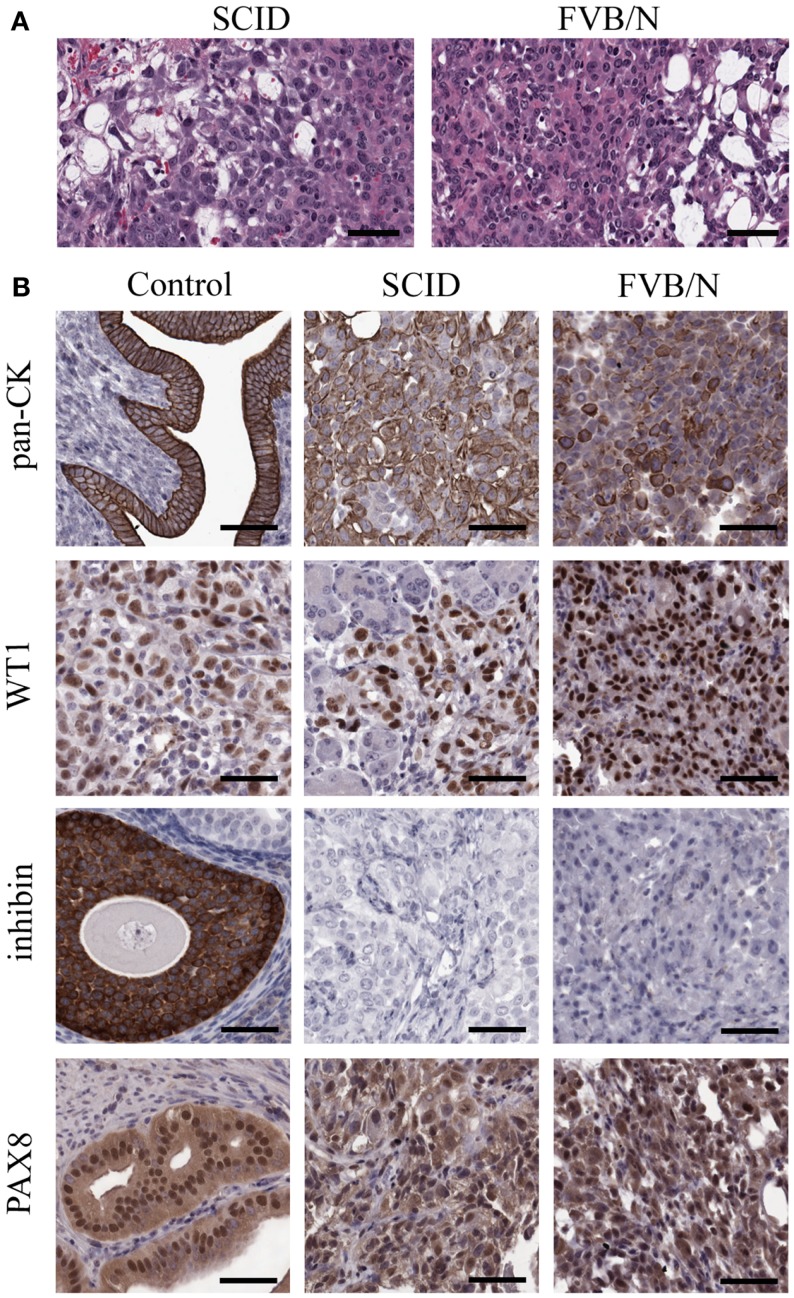
**STOSE produce high-grade serous epithelial tumors in both SCID and syngeneic FVB/N mice**. Tumors were fixed in formalin and set in paraffin blocks; 5 μm sections were used for immunohistochemistry on SCID and FVB/N STOSE cell-derived tumors. **(A)** Hematoxylin and eosin staining of STOSE cell-derived tumors. Scale bars = 100 μm. **(B)** Detection of the epithelial tumor marker, cytokeratin is presented with uterus as a positive control. Wilms tumor-1 (WT1) is a marker of HGSC and is shown with a human high-grade serous ovarian carcinoma as a control. Detection of the granulosa cell and sex-cord stromal tumor marker, inhibin, is shown with granulosa cells as a positive control. PAX8 expression is shown with a fallopian tube (oviduct) positive control. Scale bars = 50 μm.

Spontaneously transformed OSE-derived tumors from both SCID and FVB/N mice were analyzed by H and E staining for morphological classification (Figure 4A) and immunohistochemistry for expression of markers commonly found in human ovarian cancers (Figure 4B). Tumor morphology was mixed including regions of mucinous, undifferentiated, and papillary serous structures. The most common morphologies are presented in Figure 4A. To confirm an epithelial origin, tumors were stained for epithelial cytokeratins using a pan-CK antibody. Both SCID and FVB/N tumors have strong positive pan-CK staining. Wilms tumor-1 (WT1) positivity is a hallmark of HGSC (40), and all STOSE tumors stained strongly for WT1. Given the WT1 positivity, the tumors were examined for expression of another marker of HGSC, PAX8. All STOSE tumors had strong PAX8 expression. To exclude a granulosa cell origin of STOSE tumors, the expression of the granulosa cell marker inhibin was determined. No tumors expressed inhibin. Thus, STOSE-derived tumors have a pan-CK+, WT1+, inhibin−, PAX8+ profile, indicating that the STOSE tumors resemble HGSC. Since almost 100% of HGSC cases present with p53 mutations (9), DNA sequencing was performed on all 11 exons of the p53 gene in STOSE cells and no mutations were found (data not shown).

### STOSE cells retained a population of SCA1+ cells that exhibit greater malignant potential

**Figure 5:**
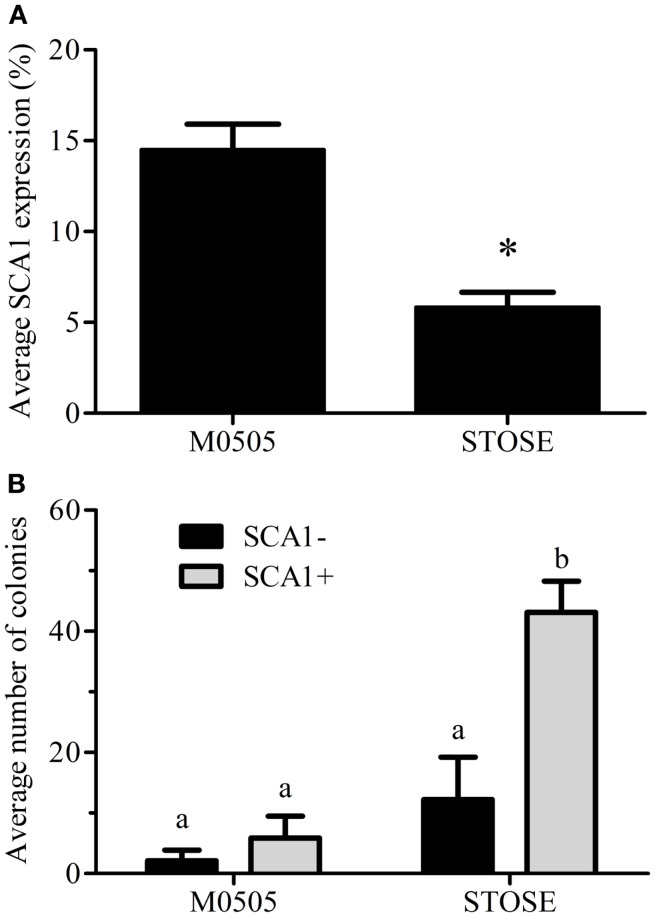
**A SCA1+ population is present in STOSE cells**. **(A)** Percentage of SCA1+ cells in M0505 (*n* = 6) and STOSE (*n* = 11) cells as assessed by flow cytometry. *p < 0.01, Student’s *t*-test. **(B)** Quantification of colony formation in soft agar by SCA1+ and SCA1− M0505 and STOSE cells. Colonies were counted using ImageJ software 7 days after seeding 2.5 × 10^4^ cells in soft agar. The average number of colonies in five fields of view is presented (*n* = 3). ANOVA was used to determine significance; bars with different letters are significantly different.

We have recently identified SCA1 as a marker of a defined stem-like population in the OSE (18). Flow cytometry confirmed that the parental M0505 cell line contains an average SCA1+ population of 14.5 ± 1.4% (*n* = 6). Interestingly, STOSE cells have retained a smaller SCA1+ population, on average 5.8 ± 0.8% (*n* = 11, Figure 5A). To determine if SCA1+ and SCA1− cells exhibit a difference in malignant potential *in vitro*, M0505 and STOSE cells were sorted for SCA1 expression and assayed for colony forming efficiency in soft agar. SCA1+ STOSE cells formed significantly more colonies than SCA1− STOSE cells (*p* < 0.05, Figure 5B).

**Figure 6:**
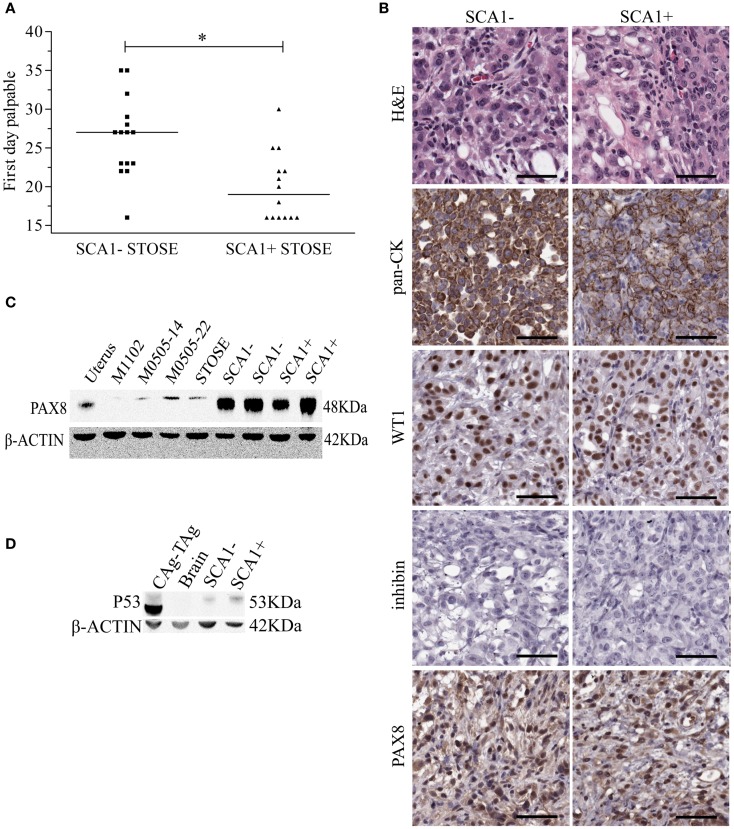
**SCA1+ STOSE cells initiate HGSC tumorigenesis faster than SCA1− STOSE cells**. Flow cytometric sorting was used to separate SCA1+ and SCA1− STOSE cells. SCA1+ (*n* = 14) and SCA1− (*n* = 15) STOSE cells were injected intrabursally into FVB/N mouse ovaries (4 × 10^4^ cells/ovary). **(A)** The first day ovarian tumors were palpable after day of injection (day 0) is presented and represents the initiation of tumorigenesis. Black lines represent median values. *p < 0.01, Student’s *t*-test. **(B)** Hematoxylin and eosin (H&E) staining and immunohistochemical staining of pan-cytokeratin (CK), WT1, inhibin, and PAX8, all commonly used markers to subtype ovarian carcinoma. Scale bars = 50 μm. **(C)** PAX8 (48 kDa) expression in cell lines and STOSE-derived SCA1+ and SCA1− tumors. Lane 1 is uterus from a wild-type FVB/N mouse as a positive control. Lane 2 is the normal M1102 OSE cell line as a negative control. Lanes 3–4 are passage 14 and 22 M0505 cells and lane 5 is STOSE cells. Lanes 6–7 and 8–9 represent tumors derived from SCA1− and SCA1+ STOSE cells, respectively. β-actin (42 kDa) was used as a loading control. **(D)** P53 (53 kDa) expression in STOSE-derived SCA1+ and SCA1− tumors. Lane 1 is a T-antigen expressing CAg–TAg tumor as a positive control. Lane 2 is brain from a wild-type FVB/N as a negative control. Lane 3–4 represents STOSE-derived SCA1− and SCA1+ tumors, respectively.

Since SCA1+ STOSE cells exhibit a more malignant phenotype *in vitro*, SCA1+ STOSE cell malignancy was tested *in vivo*. To determine if SCA1 marks cells with enhanced ability to initiate tumors, SCA1+ and SCA1− STOSE cells (4 × 10^4^) were injected IB into 29 FVB/N mouse ovaries, 15 with SCA1− cells and 14 with SCA1+ cells. SCA1+ STOSE cells initiated tumorigenesis faster than SCA1− STOSE cells with the median times to a palpable tumor of 19 (*n* = 15) and 27 days (*n* = 14), respectively (*p* < 0.01, Figure 6A). There was no difference in total tumor burden between the two groups when the mice were euthanized 116 days after STOSE cell injection, with mice having a tumor burden of 2.70 ± 0.53 g (*n* = 7) for SCA1− tumors vs. 2.72 ± 0.32 g (*n* = 6) for SCA1+ tumors. At that time point, SCA1+ and SCA1− STOSE tumors also showed a similar degree of tumor dissemination, metastasizing consistently to the uterus, stomach, diaphragm, small and large intestines, spleen, and pancreas.

To determine if the increased initiation rate in SCA1+ compared to SCA1− STOSE tumors resulted in different histological presentation, immunohistochemistry was performed using markers of HGSC. Both SCA1+ and SCA1− STOSE tumors are pan-CK+, WT1+, inhibin−, and PAX8+ (Figure 6B), with no gross histological differences evident between SCA1+ and SCA1− tumors. Western blot analysis confirmed strong PAX8 positivity in both SCA1+ and SCA1− STOSE tumors (Figure 6C), relative to the positive control, normal uterine tissue, and to the low level of expression seen in M0505 and STOSE cells cultured *in vitro*. An independent non-tumorigenic normal OSE cell line, M1102, was used as a negative control. Expression of p53 in SCA1+ and SCA1− STOSE-derived tumors was determined using western blot analysis. SCA1+ and SCA1− tumors were positive for p53 expression (Figure 6D).

## Discussion

There is substantial need for new models of HGSC that have similar expression profiles, chromosomal aberrations, and histological features characteristic of human HGSC. These models should also account for the multiple origins of HGSC in order to effectively narrow down screening targets based on the tissue of origin. The body of work presented here describes the production and characterization of a STOSE cell line. STOSE cells have lost characteristic epithelial “cobblestone” morphology, have a greatly increased proliferation rate, and form colonies in soft agar. Interestingly, there is aneuploidy in both M0505 and STOSE cells, suggesting that aneuploidy may have preceded transformation. Linear fold changes calculated from M0505 and STOSE cell microarray data revealed that STOSE cells have differentially expressed genes that are consistent with human HGSC tumor samples and previous studies on ovarian cancer cell lines. Tumors with an immunohistochemical profile of HGSC formed in all immunocompromised SCID and syngeneic FVB/N mice following IP STOSE cell injections, confirming the potential for STOSE cells to be used as a syngeneic model of HGSC. Finally, STOSE cells that express SCA1 appear to be more aggressive, with increased colony forming efficiency *in vitro* and faster tumor initiation *in vivo*.

Recent reviews have discussed the pros and cons of current models of HGSC (5, 9, 10). Current models have focused on the use of transgenics, xenografts of human cancer cells, and OSE cells transformed by genetic engineering in attempts to model HGSC. These models have had some success in modeling HGSC as well as low-grade serous, endometrioid, and granulosa cell-derived tumors, although results of these studies are highly variable and commonly have strain-dependent phenotypes (5). Most transgenics have focused on the use of the anti-Mullerian hormone type II receptor (*Amhr2* or MISIIR) promoter to drive tumor suppressor knockout or oncogene activation, but its expression in granulosa cells as well as both ovarian epithelium and fimbria can confound the results and make the origins of such cancers unclear (5). Human xenografts into immune-compromised mice have provided much knowledge on the metastasis and chemoresistance of human tumors. The lack of an immune system can limit some uses of these models, which do not accurately represent the human tumor microenvironment in which the immune system has a critical role in tumor progression and response to treatment (9, 10). Genetically engineered OSE cells have provided much insight into genes that are sufficient to transform OSE cells (41, 42), but their involvement in HGSC initiation or progression is unknown and manipulating such genes may not represent the natural progression of disease.

The STOSE cells reported here join a number of other spontaneously transformed rat (ROSE) (43, 44) and mouse OSE cell lines that have been previously reported. Syngeneic mouse models include ID8, IF5, IG10, L-MOSE, and MOSEC cells (45–48). These models are all tumorigenic in immunocompetent mice and allow the study of immunologic parameters as well as serve as a resource to test immunotherapies in ovarian cancer (48). Spontaneous models are beneficial since they arise from specific cell types, so their origins are clear (49). All of the models derived from spontaneously transformed OSE cell lines have yielded poorly differentiated epithelial carcinomas, but have not been examined further to confirm their histologic identity as it compares with human tumors. Those lines tested have shown gene expression profiles similar to human (3, 50).

The STOSE model is the first spontaneous HGSC model, as confirmed by the expression of immunohistochemical markers (pan-CK+, WT1+, inhibin−, PAX8+), consistent with human ovarian carcinomas (2, 40). The expression of WT1 and PAX8, commonly used to diagnose HGSC, help to confirm that OSE cells have the ability to spontaneously transform into HGSC. PAX8 positivity in human HGSC is one of the characteristics used to support a fimbrial origin of HGSC (2). It is well-established as a marker of fimbrial epithelium and, due to its expression in HGSC, much research has now focused on the fimbrial epithelium (2, 4). Recently, a report has shown that PAX8+ tumors can be produced from transformed hilum cells that originate in the ovary, providing additional evidence that the OSE cells can be an origin of HGSC (4). Although OSE cells have little to no PAX8 expression, our results show that both the untransformed M0505 cells as well as the STOSE cells had a low level of expression of PAX8+ (Figure 6C), suggesting that early acquisition of PAX8 expression in the M0505 cells may have facilitated the transformation of these cells. Further study of PAX8 and its function in M0505 and STOSE cells will help delineate its role in the transformation process.

The STOSE model is also the first syngeneic ovarian cancer model in the FVB/N strain of mice. All previous spontaneously transformed mouse OSE cells have been derived from C57Bl/6 mice (32, 45, 46, 48). Most spontaneous models have been produced by IP injection into syngeneic hosts, abrogating the ability to study metastasis from a specific site. The ovarian bursa is a controlled and distinct microenvironment and we have previously shown that, while tumor histology is not different when cells are injected into this location, it is an effective means to identify more invasive cells, as only aggressive cells can invade the ovary and/or breach the bursal membrane (51). Injecting cells under the bursal membrane also provides the ability to study the immune parameters associated with metastasis that could enable the production of immune therapies to prevent metastasis. The spontaneous ID8 model has produced peritoneal metastases following IB injection into their syngeneic C57Bl/6 strain of origin (52). The STOSE model also forms extensive peritoneal metastases following IB injection, making STOSE the first metastatic HGSC model in the FVB/N strain. Having spontaneous models in multiple strains is an important resource to enable investigators to show that the efficacy of a therapeutic strategy is independent of strain background, greatly improving the translation of therapeutic strategies.

Spontaneously transformed OSE cells are aneuploid and have gene expression changes consistent with human ovarian cancer. Aneuploidy is common in many cancers including ovarian cancers (19, 39, 45, 46, 53). Aneuploidy is a prognostic determinant in HGSC since severe aneuploidy is associated with poor outcome (53). STOSE cells have a high degree of aneuploidy, characterized by triploid and polyploid cells. Furthermore, the loss of genomic stability in both M0505 and STOSE cells as seen by aneuploidy may have been an early event leading to transformation that may explain the tumorigenic capacity of STOSE cells. Loss of chromosome 3, which contains many tumor suppressors, is seen in both M0505 and STOSE cells. Haploinsufficiency of chromosome 3 tumor suppressors such as *Lrrc3b* (fold change of −2.69 in STOSE cells) may underlie transformation (54). Similarly, chromosome 8 is lost in M0505 and STOSE cells and it has been shown to contain ovarian cancer susceptibility loci, allelic loss of which may have contributed to transformation (55, 56). Three down-regulated genes in STOSE cells, *Enpp2, Sfrp1*, and *Star* are all located on chromosome 8. Loss of chromosome 8 in M0505 cells may have been an early event in transformation (30, 57).

Ingenuity pathway analysis of microarray data revealed gene expression changes related to Wnt/β-catenin signaling in STOSE cells suggesting signaling in the Wnt pathway might be aberrant. Many of the down-regulated genes in STOSE cells are associated with Wnt/β-catenin signaling and have been associated with loss of heterozygosity or promoter methylation in ovarian cancer, including *Fzd4, Sfrp1*, and *Axin2* (58–61). Interestingly, *Cdkn2a* is down-regulated in 30% of HGSC cases and *Ccnd1* is amplified in 4% of the cases, according to TCGA ovarian carcinoma array (35). STOSE cells have a similar expression pattern of *Cdkn2a* and *Ccnd1*. *Cdkn2a* and *Ccnd1* are both associated with Wnt/β-catenin signaling. *Ccnd1* is a well-established target gene of β-catenin signaling and has a role in promoting cell cycle progression, while *Cdkn2a* encodes a cell cycle inhibitor that is suppressed by β-catenin (35, 62, 63). Due to the association of these two genes with human HGSC and aberrant Wnt signaling in STOSE cells, further study of the role of *Cdkn2a, Ccnd1*, and Wnt/β-catenin signaling is needed to understand the role Wnt/β-catenin signaling in the transformation of M0505 cells into STOSE cells or in the tumorigenic capacity of STOSE cells. A greater understanding of this pathway may translate to greater knowledge on the initiation and progression of HGSC.

Interestingly, *Aldh1a2* is the most down-regulated gene in STOSE cells (−170.58 fold). *Aldh1a2* is involved in retinoic acid (RA) biosynthesis and has been shown to have ubiquitous expression in the human ovarian surface epithelium (2, 29). The RA-receptor β (*Rar*β) is also down-regulated in STOSE cells (−10.80 fold) suggesting multiple aspects of RA signaling are lost. RA signaling has been shown to crosstalk with Wnt/β-catenin signaling and *Aldh1a2* has also been identified as a tumor suppressor in prostate cancer, loss of which is an early event in the disease (64, 65). Further study of *Alhd1a2*, RA signaling, and the crosstalk between RA and Wnt/B-catenin signaling may help determine the mechanisms leading to transformation and tumorigenesis in HGSC.

Investigation of a potential TIC population in the STOSE revealed that STOSE cells have retained a SCA1+ population that appears to have a more malignant phenotype than SCA1− STOSE cells. TICs have been thought to be key contributors to HGSC etiology based on the heterogeneity and recurrence that are characteristic of the disease (3, 15, 50). TICs have been identified in both human and murine ovarian cancers by sorting for CD44, CD133, CD117, CD24, ALDH1, and SCA1 expression alone or in combination (3, 15). SCA1 has also been used for the enrichment of a stem cell population in leukemia, prostate, and breast cancers (15). STOSE cells were found to contain a SCA1+ population that exhibits increased malignancy both *in vitro* as assessed by colony formation and *in vivo* as assessed by initiation of tumorigenesis. Interestingly, SCA1+ and SCA1− STOSE-derived tumors were positive for p53 expression by western blot analysis. DNA sequencing showed no mutations in the p53 gene, suggesting pathways that lead to p53 stabilization might also be aberrant in STOSE cells. Our findings that the SCA1+ population exhibits TIC characteristics is in line with a recent study on SCA1+ cells in the T2 mouse model of ovarian cancer, which showed that these cells have TIC characteristics that allow them to escape chemotherapy and produce heterogeneous tumors following treatment (15). The retention of a SCA1+ population with TIC characteristics allows us to compare tumorigenic SCA1+ STOSE cells with non-tumorigenic SCA1+ M0505 cells.

In summary, this study has led to the development of a spontaneously transformed syngeneic model of HGSC in the FVB/N mouse, the first spontaneous murine model with defined features of HGSC. The STOSE model has characteristics of human disease such as aneuploidy, gene expression, and the presence of a TIC population. This model also produces extensive metastases in the peritoneal cavity following IB injection allowing for the study of tumor dissemination. Further investigation is required to understand the contribution of Wnt/β-catenin signaling in STOSE cells. The STOSE model offers vast potential for testing of novel therapeutics, including immune therapies. This model will also allow for the discovery of new screening targets that are involved in the transition of normal cells to HGSC.

## Author Contributions

Experiments were designed by Curtis W. McCloskey and Barbara C. Vanderhyden. Olga Collins derived the cell line and performed initial validation of the transformation. All surgeries, IHC, microarray, and cell culture were done by Curtis W. McCloskey; western blotting was done by Reuben L. Goldberg; qPCR was done by Curtis W. McCloskey and Lauren E. Carter; Lisa F. Gamwell and Curtis W. McCloskey performed the flow cytometry; Kenneth Garson and Curtis W. McCloskey performed the cell cycle analysis. Elizabeth A. Macdonald and Curtis W. McCloskey performed the IP injections and necropsies. Manijeh Daneshmand performed the pathology assessment and Euridice Carmona did the IPA analysis. Curtis W. McCloskey and Ensaf M. Al-Hujaily performed DNA sequencing. The manuscript was written by Curtis W. McCloskey and Barbara C. Vanderhyden and edited by Manijeh Daneshmand and Euridice Carmona.

## Conflict of Interest Statement

The authors declare that the research was conducted in the absence of any commercial or financial relationships that could be construed as a potential conflict of interest.
